# Relationship between Residential Segregation, Later-Life Cognition, and Incident Dementia across Race/Ethnicity

**DOI:** 10.3390/ijerph182111233

**Published:** 2021-10-26

**Authors:** Daniel J. Pohl, Dominika Seblova, Justina F. Avila, Karen A. Dorsman, Erin R. Kulick, Joan A. Casey, Jennifer Manly

**Affiliations:** 1 Vagelos College of Physicians & Surgeons, Columbia University, New York, NY 10032, USA; djp2169@cumc.columbia.edu; 2 Taub Institute for Research on Alzheimer’s Disease and the Aging Brain, Vagelos College of Physicians and Surgeons, Columbia University, New York, NY 10032, USA; ds3868@cumc.columbia.edu (D.S.); jfa2125@cumc.columbia.edu (J.F.A.); 3 Gertrude H. Sergievsky Center, Vagelos College of Physicians and Surgeons, Columbia University, New York, NY 10032, USA; 4 Department of Neurology, Vagelos College of Physicians and Surgeons, Columbia University, New York, NY 10032, USA; erin.kulick@temple.edu; 5 Department of Psychiatry, The University of Texas Southwestern Medical Center, Dallas, TX 75235, USA; karen.dorsman@utsouthwestern.edu; 6 Department of Epidemiology and Biostatistics, College of Public Health, Temple University, Philadephia, PA 19122, USA; 7 Department of Environmental Health Sciences, Mailman School of Public Health, Columbia University, New York, NY 10031, USA; jac2250@cumc.columbia.edu

**Keywords:** racial/ethnic residential segregation, health inequity, cognition, structural racism

## Abstract

Systemic racism leads to racial/ethnic residential segregation, which can result in health inequities. We examined if the associations between residential segregation and later-life cognition and dementia differed based on segregation measure and by participant race/ethnicity. Tests of memory (*n* = 4616), language (*n* = 4333), visuospatial abilities (*n* = 4557), and incident dementia (*n* = 4556) were analyzed in older residents of Northern Manhattan, New York (mean age: 75.7 years). Segregation was measured at the block group-level using three indices: dissimilarity, isolation, and interaction. We fit multilevel linear or Cox proportional hazards models and included a race/ethnicity × segregation term to test for differential associations, adjusting for socioeconomic and health factors. Living in block groups with higher proportions of minoritized people was associated with −0.05 SD lower language scores. Living in block groups with higher potential contact between racial/ethnic groups was associated with 0.06–0.1 SD higher language scores. The findings were less pronounced for other cognitive domains and for incident dementia. Non-Hispanic Black adults were most likely to experience negative effects of neighborhood segregation on cognition (language and memory) and dementia. All indices partly capture downstream effects of structural racism (i.e., unequal distributions of wealth/resources) on cognition. Therefore, desegregation and equitable access to resources have the potential to improve later-life cognitive health.

## 1. Introduction

Ecosocial factors, such as inadequate health care, socially inflicted trauma (e.g., racism), and economic and social deprivation, influence health [1]. Racial/ethnic residential segregation (hereafter racial/ethnic segregation), defined as uneven non-random distribution of minoritized groups among neighboring residential areas [2], is an important ecosocial factor. In the United States, racial/ethnic segregation reflects a long history of policies, such as redlining, that have kept access to resources restricted for minoritized groups [2]. There are, therefore, spatial differences in access to resources that are important for health. These health-linked resources include access to land and property, quality education, jobs, transportation, and a clean environment with green spaces, in addition to freedom from police brutality, racism and bias, and other stressors [2,3,4]. Consequently, racial/ethnic segregation has been linked to many health outcomes, from adverse birth outcomes [5,6] to diabetes [7] and hypertension [8] to death [9]. Residential environment has also been linked to life-style factors, such as physical activity [10], which in turn are associated with cognitive health. However, there is limited evidence regarding the relationship of racial/ethnic segregation and later-life cognitive health [11,12,13].

Two main pathways have been evoked to explain the impact of racial/ethnic residential segregation on cognition. First, segregated areas with residents from predominantly minoritized groups suffer from higher resource deprivation. For example, living in an area with high levels of poverty is associated with higher rates of obesity and cardiovascular disease [14,15] possibly due to lower access to quality nutrition. In turn, obesity and cardiovascular disease are associated with an increased risk of poor later-life cognition [11]. Members of the Black community have been especially affected by the unequal and unjust distribution of wealth, resources, and opportunities due to structural racism [16]. In line with this pathway, a study focusing on non-Hispanic (NH) Black older adults indicated that greater cumulative exposure to living in segregated census tracts was associated with a worse cognitive scores [17]. Second, social cohesion, which is frequently increased in segregated minority areas and especially in ethnic enclaves, could positively affect cognition [18], for example, by increasing social interaction and reducing loneliness. The role of social cohesion has been highlighted especially for Latinx communities [19,20].

In this study, we examine residential segregation in the Washington Heights/Inwood area in Northern Manhattan, New York. The area is characterized by racial/ethnic diversity with approximately 70% of the residents self-identifying as Hispanic, 17% as NH White, and 8% as NH Black [21] and is home to one of the most prominent Dominican American enclaves in the United States. The extant literature on the role of residential segregation and cognitive health for members of the Latinx community is mixed. Aneshensel et al. (2011) found that living in predominantly Hispanic neighborhoods was not protective for cognitive functioning [22], but Kovalchik et al. (2015) reported opposite findings [23]. Further, studies examining Mexican American enclaves in the United States also arrived at opposite conclusions with one indicating that living in an enclave was associated with higher risk of cognitive impairment [16], while the others reported no differences [24] or better cognitive outcomes [15] for enclave residents. Nativity and country of origin have been indicated as important predictors of health among the pan-ethnic Latinx group [25]. These findings indicate that there may be a differential balance of resource deprivation and social cohesion mechanisms based on race/ethnicity and nativity. In sum, it is key to examine how the association of residential segregation and cognition differs by race/ethnicity.

Uncovering the association between segregation and cognition hinges on the ability to measure segregation. The two key dimensions to consider are (1) potential for contact and (2) level of clustering [26]. Measures of potential for contact include isolation and interaction indices. Briefly, the greater the value of the isolation index, the less likely one is to come into contact with someone of a different racial/ethnic group. The converse is true for the interaction index [26]. These indices may more adequately capture benefits of social contact, corresponding to the social cohesion pathways. Measures along the clustering dimension, such as dissimilarity, might better capture inequitable access to resources [27]. The nascent literature regarding residential segregation and cognition lacks comparative examination across multiple indices—in other words, we are not yet sure which of these indices are most important for cognitive health. Hence, we included three indices—isolation, interaction, and dissimilarity—in our study. 

The aim of the current study was to examine how racial residential segregation relates to cognitive abilities and incident dementia in older individuals from a large representative cohort in Northern Manhattan, New York. Specifically, we examine: (1) the cross-sectional association between racial/ethnic residential segregation and later-life cognitive abilities (memory, language, and visuospatial abilities) and incident dementia; (2) if the association differed based on measure of segregation; and (3) how the associations differed across individual-level race/ethnicity. We hypothesized that living in areas with higher segregation will be negatively associated with one’s cognitive health (i.e., lower cognitive scores and higher incident dementia), and that the association will be most pronounced among those from minoritized racial and ethnic groups. We had no a priori hypothesis regarding individual cognitive domains.

## 2. Materials and Methods

### 2.1. Source Population and Analytic Sample

Participants came from the Washington Heights Inwood Columbia Aging Project (WHICAP). WHICAP is a longitudinal, prospective cohort recruiting community-residing Medicare recipients 65 years and older in Northern Manhattan. There is substantial racial/ethnic segregation in the studied area with NH Black individuals residing predominantly in the southeastern part of the neighborhood. Since 1980, the neighborhood has been home to one of the most prominent Dominican American enclaves in the United States. Therefore, the area provides a unique setting in which to examine the role of residential segregation in a diverse population.

Recruitment for the WHICAP study occurred in three waves (1992, 1999, and 2009) and each participant completed detailed neuropsychological assessments either in English or Spanish and provided demographic, functional, and medical information. The WHICAP’s design and methods have been described in detail elsewhere [28]. For the current analyses, we considered WHICAP respondents with at least one visit who self-identified as NH White, NH Black, or Hispanic (*n* = 6157). This resulted in exclusion of 97 individuals. We also excluded 240 individuals from analyses due to missing residential address data. Further, those missing data on one or more covariates (*n* = 1224; 19.9%, see Supplementary Figures S1 and S2) were excluded, leaving 4616 participants (mean age: 75.7; SD 6.5 years) for the memory analysis, 4333 in the language analysis (mean age: 75.7; SD 6.5 years), and 4557 in the visuospatial abilities analysis (mean age: 75.7; SD 6.4 years). Finally, we excluded those with prevalent dementia diagnosis (*n* = 837), leaving 4556 participants in the analysis of incident dementia (mean age: 75.7; SD 6.5). Those with missing covariates were more likely to reside in segregated areas, had lower education, were more likely NH Black or Hispanic, were substantially older (79.0 vs. 75.8 years) and had lower cognitive scores but less frequent incident dementia than those without missing covariates (Supplementary Table S1). The impact of missingness on our findings was examined using multiple imputation.

### 2.2. Effect Modifier: Race/Ethnicity

Self-reported race/ethnicity was obtained in two steps. All WHICAP respondents were first asked to report their racial group. Then, in a second question, participants indicated whether they were of Hispanic origin. For the analyses, individuals were categorized into NH White, NH Black, and Hispanic groups.

### 2.3. Exposure of Interest: Racial Residential Segregation from 2005–2009

**Figure 1:**
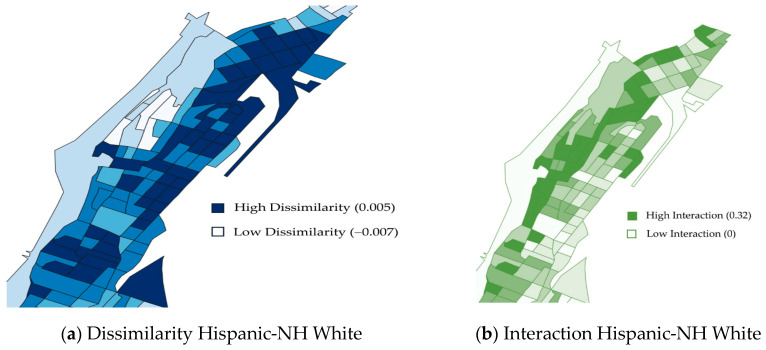

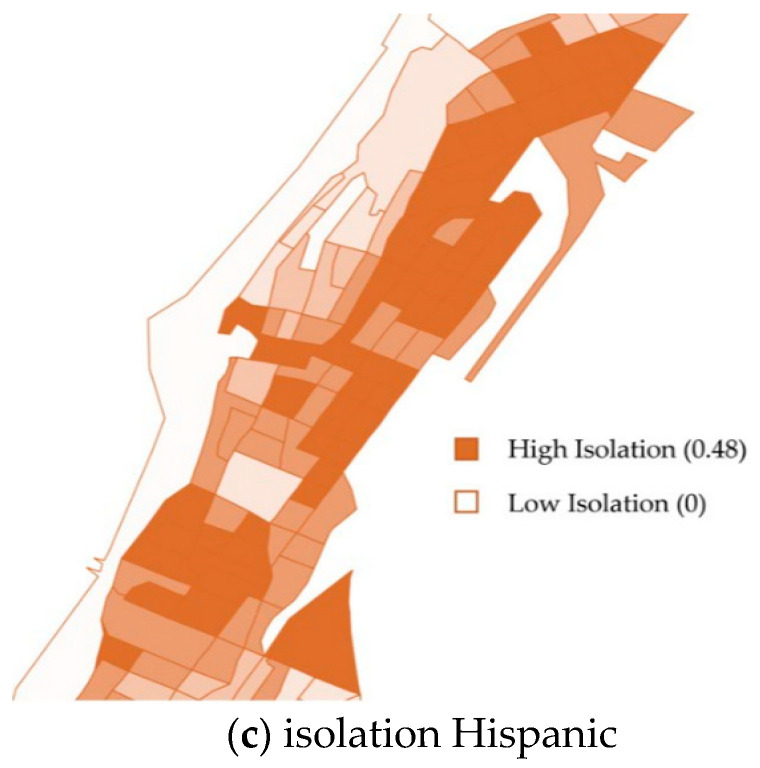
This figure demonstrates the unique information provided by the three different racial residential segregation indices (dissimilarity, interaction, isolation) using the Hispanic group as an example. The maps above show the block groups for Washington Heights and Inwood, New York. (**a**) Dissimilarity index for people who are Hispanic and non-Hispanic White. Darker blue is higher dissimilarity; lighter blue is lower dissimilarity. (**b**) Interaction index for people who are Hispanic and non-Hispanic White. Darker green is higher interaction; lighter green is lower interaction. (**c**) Isolation index for people who are Hispanic. Darker orange is higher isolation; lighter orange is lower isolation. Maps for other 4 indices are provided in Supplementary Figure S3.

We derived three previously described [26,29,30,31] indices (dissimilarity, isolation, and interaction; Figure 1) capturing residential segregation from the 2005 to 2009 American Community Survey (ACS). It is important to note that the studied area has very high residential density and racial/ethnic composition can vary significantly from block to block. Thus, in contrast to previous studies that defined segregation at the census tract level, the most common unit of analysis [32], we operationalized residential segregation at the census block groups, which are smaller (typically 600–30,000 people) and more adequately capture the residential context in Washington Heights/Inwood. WHICAP participants’ addresses obtained at their baseline visit (1992, 1997, or 2009) were geocoded and matched to segregation indices using block group identifiers. The 2005–2009 ACS data are the earliest available, however 90% of the WHICAP respondents did not change their residence. Respondents in our analyses lived in 345 block groups.

Each of the examined indices brings out a different aspect of residential segregation (level of clustering and contact potential). Dissimilarity captures clustering and a smaller value on this index indicates more homogeneous distribution of a particular racial/ethnic group across the areal unit. In other words, low dissimilarity values equate to less segregated areas. Isolation and interaction are both measures of potential contact. When comparing only two racial/ethnic groups, interaction and isolation are the inverse of each other; however, because there are multiple racial/ethnic groups in each areal unit in our study, interaction and isolation provide unique information (Figure 1). In our study we calculated three isolation measures—one for NH White, one for NH Black, and one for Hispanic respondents—two interaction, and two dissimilarity measures (NH White-NH Black and NH White-Hispanic).

Segregation indices typically aggregate smaller units (block groups) into larger ones (census tracts) to compare individual census tracts to the overall population distribution in the studied area. We forewent the aggregation process and calculated the indices exclusively at the level of a block group. We decided on this approach because of the large variability in residential composition within census tracts in New York City. For example, for the dissimilarity index, our operationalization captures the number of people who would need to move to create an equal proportion of people of the studied races/ethnicities in a block group. Our operationalization does not allow for relative comparisons that would indicate if a given block group is more or less segregated than other block groups in the Washington Heights and Inwood area. Rather, our calculations (see Supplementary Materials S1) allow us to identify each unit as either segregated or not segregated. For all indices, we used the mean value in our sample as a cut-off when defining segregated block groups. For dissimilarity and isolation, values greater than the mean indicate more segregated areas. In contrast, for interaction, values above the mean indicate lower segregation.

### 2.4. Outcomes: Cognitive Performance and Incident Dementia

Cognitive domains of memory (*n* = 4616), language (*n* = 4333), and visuospatial abilities (*n* = 4557) were derived from baseline neuropsychological tests administered either in Spanish or English, based on previously published confirmatory factor analysis [33,34]. Briefly, memory scores combined the total recall, delayed recall, and delayed recognition components of the Selective Reminding Test [35]. Language abilities were measured by total naming (Boston Naming Test), letter and category fluency, and similarities (Wechsler Adult Intelligence Scale-Revised), repetition, and comprehension (Boston Diagnostic Aphasia Evaluation). The visuospatial abilities score included the recognition and matching test of the Benton Visual Retention Test [36], the Rosen Drawing Test [37], and the Identities and Oddities subtest of the Mattis Dementia Rating Scale [38]. The battery has been adapted to Spanish [39]; specifically, the interview questions, instructions, and stimuli were translated into Spanish by a committee of Spanish speakers from areas represented in the Washington Heights area (Cuba, Puerto Rico, and Dominican Republic). Next, material was back-translated to ensure accuracy. When necessary, scoring criteria were modified to allow credit to be given for responses reflecting regional idioms. Interviews of Spanish-speaking participants were conducted by bilingual staff [33]. This battery of neurocognitive assessments was validated in individuals of varying educational attainment and linguistic abilities [33,40] and the composite scores were found to be invariant across race/ethnicity [34] and language of testing [33]. Scores for each task were converted to z-score metric using the mean and SD for all WHICAP participants at the initial visit. Subsequently, domain scores were computed by taking the weighted average of the z-scores of all relevant tasks (6 tasks for language abilities, 3 tasks for memory, 4 tasks for visuospatial abilities).

Diagnosis of dementia was established by a team of neurologists, psychiatrists, and neuropsychologists based on a review of available clinical information, including neuropsychologic and medical patient data (not including radiological data) gathered at that visit. Follow-up visits occur at 18–24 months intervals for up to 23 years. The team was blind to scores and diagnoses at prior visits. The criteria for all-cause dementia diagnosis was based on standard research criteria [41], and then dementia subtype was determined based on research criteria for probable or possible Alzheimer’s disease [41], Lewy body dementia [42], or vascular dementia [43]. This approach has been validated in this population [44]. We operationalize dementia as a binary indicator of having any dementia diagnosis. Age at the visit when a dementia diagnosis was made was used as the timing of incident dementia.

### 2.5. Other Variables

All models included age at baseline. A binary indicator for sex/gender was derived based on self-identification as male or female. We considered several socioeconomic variables: own level of education (years), occupation, and childhood socioeconomic status (cSES). CSES measure was comprised of parental education and occupation. Birthplace was classified as USA, Puerto Rico, Dominican Republic, or unreported. We included an ordinal variable indicating recruitment cohort (1992, 1999, or 2009) to adjust for birth cohort and differences in recruitment over time. An indicator for language of neurocognitive test administration (English/Spanish) was also considered. Cardiovascular health is related to cognition [45]; thus, we included a count of self-reported cardiovascular diseases, namely, hypertension, diabetes, heart disease, and stroke (range 0–4). Finally, we included the proportion of the block group living below the federal poverty line (range 0.0–0.88). All participant demographics used as covariates were collected at baseline.

### 2.6. Statistical Analysis

**Figure 2:**
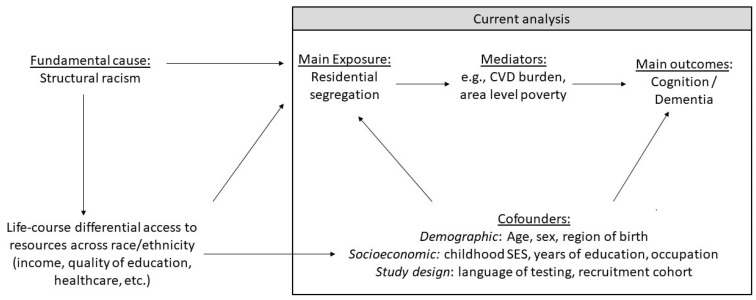
A conceptual and analytical model of the relationships studied. Residential segregation was measured by 6 indices at the census block group level. To test if the association of residential segregation varied based on respondent race/ethnicity, effect modification was examined by including individual-level race/ethnicity × segregation term.

The association of each segregation index and later-life cognitive performance was estimated using multilevel linear models. Our conceptual and analytical model is presented in Figure 2. For each segregation index and cognitive composite, we fit three models. Model 1 included age, sex/gender, and race/ethnicity. Model 2, which we consider our main specification, added possible confounders of the examined association, namely, cSES, years of education, occupation, language of test administration, birthplace, and recruitment cohort. Model 3 added possible mediators of the association—cardiovascular disease event count and neighborhood poverty level. To test if the association of residential segregation varied based on respondent race/ethnicity, we fit the same set of models but included an individual-level race/ethnicity × segregation term. We considered interactions with *p* < 0.2 as indicating differential association because statistical power to detect interactions is typically lower than the power needed to detect main effects [46]. The same modeling approach was adopted for incident dementia, using Cox proportional hazards regression models with random effects at the block group-level (so called shared frailty models) [47]. Age was used as the underlying time scale, and individuals were followed up until dementia diagnosis, death, or loss to follow-up. In a sensitivity analysis, we re-estimated our main models using multiple imputation employing Markov chain Monte Carlo (MCMC) method with 10 imputations to examine the role of missing data on our findings.

Our data is spatial in nature with respondents clustered within block groups; therefore, our multilevel regression models included random effects for the block group to take into account clustering. We also examined the need for employing more advanced spatial models (e.g., spatial error or lag models) by checking the extent of spatial autocorrelation [48] based on regression residuals using Moran’s I statistic. The results indicated that residual spatial autocorrelation was handled appropriately via block group random effects.

Data were analyzed using R Studio and SAS 9.4. Maps of segregation indices and geographic racial/ethnic composition were generated using QGIS.

## 3. Results

### 3.1. Descriptive Statistics

The majority of our sample self-identified as Hispanic (44.6%; *n* = 2095, mean age: 75.8), followed by NH Black (30.4%; *n* = 1425, mean age: 75.8) and NH White (25.0%; *n* = 1173, mean age: 75.8). Segregated areas had higher proportions of Hispanic (50.8% vs. 36.9%) and NH Black respondents (36.0% vs. 23.3%) than non-segregated areas (Table 1) and nearly 30% of those living in segregated areas were born in the Dominican Republic. In general, those living in segregated block groups had lower levels of education, lower occupation, and higher rates of CVD comorbidities (Table 1). Overall, those living in segregated areas scored lower on cognitive tests and had higher dementia incidence (*n* = 412, 18.9% vs. *n* = 325, 13.7% in non-segregated areas). The follow up period for those living in segregated areas was on average 5.1 years (4.8 SD) and 5.5 years (4.8 SD) for those living in non-segregated areas.

There was variation in resource distribution across high and low segregation block groups and segregation measure (Supplementary Table S2). Isolated NH White areas had the lowest proportion of residents living below the poverty line (mean: 0.12; SD: 0.09; range: 0.0–0.4), followed by desegregated areas (high NH Black-NH White and Hispanic-NH White interaction). Areas with predominantly minoritized backgrounds had the highest proportion of residents living below the poverty line (e.g., high NH Black isolation; mean: 0.28; SD: 0.12; range: 0.0–0.9).

### 3.2. Block-Group Segregation and Level of Later Life Cognitive Abilities

All segregation indices were associated with language abilities in our main models (Table 2). Specifically, living in segregated areas with a higher proportion of people from minoritized backgrounds was associated with about −0.05 SD lower score (Table 2). Living in desegregated areas with higher potential contact between racial/ethnic groups (high interaction) or in areas with higher proportion of NH White adults (high isolation) was associated with about 0.06–0.1 SD higher language scores (Table 2). For comparison, one year of age was associated with 0.02 SD lower scores, thus living in desegregated areas corresponds to 3–5 years (= 0.06/0.02–0.1/0.02) difference in language test scores. Individual level socioeconomic confounders reduced minimally adjusted estimates on average by 31% (see Supplementary Table S3). The addition of mediators (Supplementary Table S4) attenuated the associations on average by an additional 15%. For all but one index (NH Black isolation), the estimates and their respective confidence intervals did not include the null value. Both the proportion of the block group below the poverty line and CVD count were associated with language scores (data upon request). After accounting for missing data, the estimates for two indices using Hispanic adults as the reference (Hispanic-NH White dissimilarity, and Hispanic isolation) were substantially reduced (Supplementary Table S5).

For other cognitive domains, the associations were less pronounced, but similar pattern as for language domain was clear for segregation indices using NH Black adults as a reference (Supplementary Table S5). In summary, living in areas with a higher proportion of NH Black adults was associated with about −0.05 SD lower scores on the memory domain (Table 2 and Table S5) and living in areas with higher potential interaction between NH Whites and Hispanics was associated with 0.06 SD higher memory performance (Table 2 and Table S5). The overall pattern of findings for visuospatial skills was similar to language abilities: living in areas with a higher proportion of people from minoritized backgrounds was associated with lower scores (≈−0.04 SD, Table 2) and living in areas with a higher proportion of NH Whites or with higher potential contact between racial/ethnic groups was associated with higher scores (≈0.01 to 0.05 SD, Table 2). Accounting for missing data reduced the estimates for segregation indices using Hispanic adults as a reference. Estimates and confidence intervals for two indices using NH Black adults as a reference (NH Black-NH White dissimilarity, and NH Black isolation) did not include the null in our sensitivity analyses (Supplementary Table S5).

**Figure 3:**
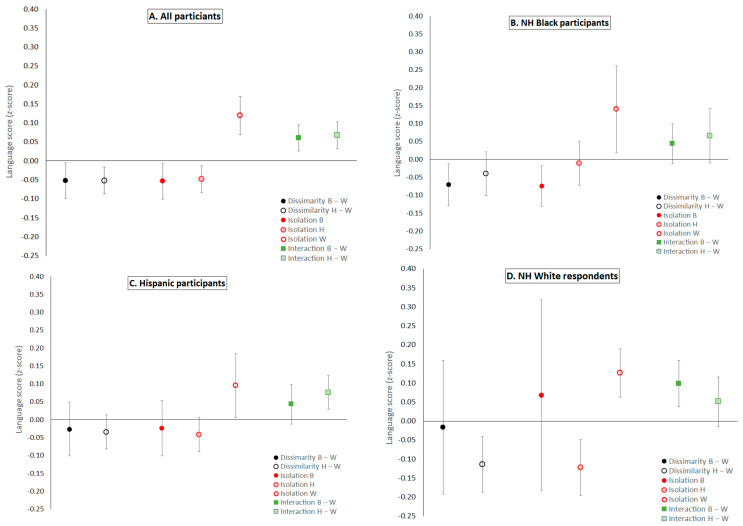
Association of residential segregation indices and language scores for the whole sample (**A**) and three race/ethnicity groups (**B**–**D**), which were derived from models with a segregation index x ace/ethnicity interaction (estimates in Table 2 and Table 3). The models controlled for age, sex/gender, race/ethnicity, childhood socioeconomic position, years of education, occupation, language of test administration, birthplace, and recruitment cohort. For all indices, we used the mean value within our sample to create binary indicators of segregated census blocks. Higher dissimilarity and isolation indicate a block group with more people from minoritized backgrounds, whereas higher interaction indicates more desegregated areas. Note: confidence intervals for effect of living in census blocks where the majority of residents are NH Black adults (isolation B and dissimilarity B-W) on NH White respondents (panel **D**) have large confidence intervals due to small sample sizes (*n* = 14 and *n* = 43, respectively).

Few associations differed across race/ethnicity based on interaction terms in our main models (Hispanic isolation for the language domain and dissimilarity NH White-NH Black for the visuospatial domain; Table 3) and showed that the negative association between living in segregated areas and cognition was largest for NH Whites (≈−0.1 SD). Similarly, the Hispanic-NH White dissimilarity index showed larger estimated effects on cognition among NH Whites (Table 3 and Figure 3) but based on the *p*-value for the interaction term, the differences were not statistically reliable (Supplementary Table S6). The number of NH Whites living in areas with predominantly minoritized residents was small. The association of living in areas with a higher proportion of NH Black respondents and lower cognitive function was reliable only for NH Black respondents for language abilities (≈−0.07 SD for dissimilarity NH Black-NH White and NH Black isolation, Table 3 and Figure 3) and memory (≈−0.07 SD for dissimilarity NH Black-NH White, Table 3 and Figure 3). Living in areas with higher potential contact with other racial/ethnic groups was associated with better language abilities for all racial/ethnic groups but was only reliable for NH Whites (interaction NH White-NH Black) and Hispanic (interaction Hispanic-NH White) respondents. Most isolation and segregation indices were reliably associated with the visuospatial domain for Hispanic respondents, where areas with a higher proportion of minoritized population predicted lower scores.

### 3.3. Incident Dementia

In models adjusting for socioeconomic confounders, the results indicated that living in areas with higher proportion of people from minoritized background was associated with higher dementia incidence (Table 2). People living in areas with predominantly NH White residents had a lower risk of incident dementia. While the confidence intervals in main analyses included the null, after accounting for missing data, living in areas with predominantly NH Black residents was consistently associated with increased dementia risk (dissimilarity NH Black: HR 1.25; 95% CI: 1.01, 1.54 and isolation NH Black: HR 1.29; 95% CI: 1.05; 1.57). These results were driven by estimates for NH Black respondents (Table 3; dissimilarity NH Black: HR 1.70; 95% CI: 1.34, 2.27 and isolation NH Black: HR 1.72; 95% CI: 1.32, 2.24).

## 4. Discussion

The aim of this study was to examine the relationship of racial/ethnic residential segregation with cognitive ability and incident dementia, and to determine if these associations varied by race/ethnicity and segregation index. To do this, we leveraged a large ethnically/racially diverse cohort in Northern Manhattan, New York. One of the most consistent results in our study was that NH Black older adults living in segregated Black neighborhoods had poorer performance on measures of language, memory, and higher risk of developing dementia, corresponding to their unequal and unjust high exposure to resource deprivation as a consequence of structural racism. The negative association of residential segregation and cognition was also replicated in the whole sample. On the other hand, living in more desegregated areas that are more affluent was associated with higher cognitive test scores for all groups. The observed associations were most consistent and strongest for the language domain, and the magnitude of these associations was comparable to the effect of 3–5 years of chronological age on cognitive ability.

We replicated the findings obtained by Caunca et al. (2020), that older NH Black people living in under-resourced segregated NH Black neighborhoods had lower language and memory test scores and were at higher risk of dementia than NH Black residents living in more affluent racially desegregated areas [17]. Emerging literature has linked residence in disadvantaged neighborhoods with compromised brain health, including cortical thinning in Alzheimer’s disease signature regions, but this was studied in a predominantly NH White sample [19,22,23,49]. Thus, we extend the literature by providing evidence of the role of segregation on pathological aging in the form of dementia in non-White populations.

An important contribution of our study is that we examined multiple segregation indices in a diverse neighborhood. Dissimilarity has been proposed as an indicator of inequitable distribution of resources [27]. The exposure indices—interaction and isolation—are thought to at least partly reflect mechanisms reliant on social contact, such as support from one’s social network, adoption of health-related behaviors [50] or exposure to stressors, such as discrimination [51,52]. There were clear differences in resource distribution across different types of areas in our study and segregation measure. Proportion of residents living below the poverty line was about 55% lower in isolated White areas and 10% lower in racially/ethnically desegrated areas, compared to segregated areas. Overall, our findings indicate that living in more affluent areas was beneficial for cognition, and the effect was most pronounced with the NH White isolation index. For all other indices, we found a similar magnitude of associations, suggesting that their impact on cognitive aging and dementia risk most likely reflects similar mechanisms. In sum, we believe all of the residential segregation indices predominantly capture downstream effects of structural racism, such as unequal distributions of wealth, resources, and opportunity.

Previous studies have explored the association between residential segregation and overall cognition across census tracts [19,22,23]. However, fewer studies have examined this relationship within specific cognitive domains [17]. Our study showed that residential segregation—living in under-resourced segregated NH Black neighborhoods—was associated with worse cognitive scores and incident dementia, suggesting minimal differences across domains. The observed relationships were most consistent for the domain of language, where living in under-resourced areas was associated with lower scores. Language abilities are largely dependent on formal education, but in our study these relationships remained in models adjusting for years of education and other SES confounders. Compared to memory or visuospatial abilities, tests of language abilities rely to a greater extent on culturally specific skills, knowledge, and stimuli, often reinforced during schooling. Our findings indicate that residential segregation independently contributes to these life-course exposures, most likely through cumulative differences in opportunity structures [53], such as access to quality education [54]. For example, in the US, public schools are funded by property taxes, which can result in inequitable educational experiences where poor areas have less money to provide to their schools. Pattern of findings across domains and further examination of differences across cognitive subdomains is warranted.

Individual- and neighborhood-level socioeconomic factors are thought to be important mediators linking residential segregation and cognition [55,56,57,58], as living in more segregated Black and Hispanic areas restricts access to social, economic, and political resources. Block group poverty level was associated with scores on tests in the language domain (but not other cognitive outcomes). While the association between residential segregation and cognition was substantially reduced when individual socioeconomic factors were included in our models, the estimates and their respective confidence intervals did not include the null, indicating that residential segregation may capture additional aspects of exposure to cumulative disadvantage. These additional aspects may include negative psychosocial effects of increased lifelong exposure discrimination, more limited access to health care, and environmental exposures (e.g., air, noise, and water pollution, access to green spaces) [2]. Adjusting for concurrent depressive symptoms, another possible mediator of the studied relationship, did not alter our findings (Supplementary Table S6). Furthermore, the persistence of associations after adjustment for individual socioeconomic factors points to the importance of societal intervention, such as resource redistribution, as opposed to individual-level changes.

Previous research proposed that living in segregated ethnic enclaves is a protective factor because of stronger social networks and the support they provide [19,20,59,60]. The Washington Heights area is home to a Dominican American enclave, thus we believe protective effects of living in an enclave would be evident in estimates for the Hispanic respondents. We believe that the Hispanic isolation index should best capture the “enclave effect”. Specifically, if living in an ethnic enclave is protective for later-life cognition, we would see that the effect of Hispanic isolation among Hispanic respondents would be smaller than the effect of Black isolation for NH Black respondents. However, our findings indicate that isolation was negatively associated with cognition in both racial/ethnic groups, replicating findings by Sheffield and Peeks (2009) and providing further support for the resource deprivation, rather than social network, pathway. Nevertheless, this does not mean that living in an ethnic enclave does not provide access to community support and other important resources for minoritized populations. Instead, it indicates that the compounded disadvantages [61] resulting from racist practices faced by individuals in segregated communities may erase the cognitive benefits [22] of increased social resources. Such trade-offs between resource deprivation and social support should be explicitly examined in the future.

The strengths of this study include the consideration of multiple indices of segregation at the block group level, examination of multiple cognitive domains based on validated neurocognitive measures, longitudinal assessment of incident dementia risk, large sample size, and examination of heterogeneous effects by race and ethnicity. However, the study also has a number of limitations. First, our study design did not allow us to determine the causal direction of the relationship between residential segregation and cognitive function or dementia risk. Second, there is a potential for exposure misclassification since respondents’ addresses used in this analysis were obtained at their baseline (1992, 1997, or 2009), but segregation indices were derived from the earliest available block group-level ACS statistics, averaged between 2005 and 2009. Nevertheless, the majority (90%) of the WHICAP participants did not change their residence since the baseline assessment, suggesting that misclassification due to mobility is likely limited. Third, the composition of the block groups may have changed between the time of the baseline study visit and the ACS data collection due to ongoing gentrification [62]. For earlier WHICAP recruitment cohorts (1992 and 1997), segregation was higher, meaning we may have underestimated the positive “enclave effect” as well as the effect of segregation through resource deprivation. Fourth, 19.9% of respondents were missing data on covariates, especially childhood SES, and were excluded from the study. Those with missing covariates were more likely to live in segregated areas and had lower SES and cognition. Thus, our estimates likely underestimate the association between residential segregation and cognition. Fifth, despite the heterogeneity of Hispanic, Black, and White populations, we used single pan-racial/ethnic categories. Previous studies suggest that within-group heterogeneity may act as a modifier of the relationships studied in this paper and should be a topic of future research [19,22,24]. Sixth, while many contextual factors may be important for cognitive health, we chose to consider only poverty level. Nevertheless, we conceptualize these factors as possible mediators of the studied relationship. Longitudinal studies using mediation analysis are needed to clarify the role of these factors. Seventh, while we account for several confounders, we cannot rule out the possibility that unmeasured confounding, for example, by family history of dementia, neurological or neuropsychological disorders [63,64], biased our findings. Nevertheless, many of these factors (e.g., substance abuse) are relatively rare, making them unlikely drivers of our observed relationships.

## 5. Conclusions

Residential segregation is a consequence of structural racism, is maintained through unequal and unjust distribution of wealth, resources, and opportunities, and is a consistent predictor of poor health outcomes among Black people [16]. Our study of older adults in Northern Manhattan, New York, suggested that (1) Black and Hispanic older adults were more likely to live in segregated neighborhoods that had twice the proportion of residents living below the poverty line; (2) residential segregation was associated with decreased cognitive function and increased dementia risk even after taking into account individual factors; and (3) living in more resource-rich, desegregated neighborhoods was positively associated with later-life cognitive health. Our results suggest that policies that reduce residential segregation, such as access to affordable housing, and resource redistribution to low-income communities have the potential to narrow cognitive health disparities among Black and Hispanic older adults. With aging Baby Boomer generation and increasing dementia prevalence and costs [65], neighborhood-level interventions should be considered to achieve brain health equity and maintain the wellbeing of society.

## Figures and Tables

**Table 1:** Sample characteristics at baseline stratified by racial residential category.

	Racial Residential Segregation ^1^	
Characteristic	Segregated *n* = 2603 (56.4%)	Not Segregated *n* = 2013 (43.6%)
Age, mean (SD), (y)	75.91 (6.44)	75.52 (6.47)
Educational level, mean (SD), (y)	9.21 (4.80)	11.42 (4.98)
Women, No. (%)	1804 (69.30)	1310 (65.08)
Race/ethnicity, No. (%)		
Hispanic/Latino	1322 (50.79)	742 (36.86)
Non-Hispanic Black	938 (36.04)	468 (23.25)
Non-Hispanic White	343 (13.18)	803 (39.89)
Tested in English, No. (%)	1348 (51.79)	1337 (66.42)
CSES, mean (SD)	−0.091 (0.644)	0.083 (0.653)
Occupation, No. (%)		
Low	1511 (58.05)	823 (40.88)
Middle	543 (20.86)	454 (22.55)
High	424 (16.29)	648 (32.19)
Not reported	125 (4.80)	88 (4.37)
Birthplace, No. (%)		
USA	910 (34.96)	810 (40.24)
Puerto Rico	119 (4.57)	70 (3.48)
Dominican Republic	794 (30.50)	347 (17.24)
Not reported	780 (29.97)	786 (39.05)
Cohort, No. (%)		
1992	679 (26.09)	505 (25.09)
1998	964 (37.03)	717 (35.62)
2009	960 (36.88)	791 (39.29)
Area poverty level, mean (SD)	0.267 (0.118)	0.212 (0.130)
CVD count, mean (SD)	1.240 (0.932)	1.088 (0.927)
Cognitive scores ^2^, mean (SD)		
Memory	0.009 (0.821)	0.245 (0.850)
Language	−0.033 (0.700)	0.299 (0.769)
Visuospatial abilities	0.054 (0.687)	0.327 (0.657)
Incident dementia ^2^, No. (%)	412 (18.9)	325 (13.7%)

^1^ Segregation classification for the descriptive table was determined by combining all seven segregation indices in the following manner: segregated area was area with low interaction and high dissimilarity and high isolation; ^2^ note that the sample sizes vary for each outcome; abbreviations: cSES = childhood socioeconomic status; CVD = cardiovascular disease.

**Table 2:** Results of multilevel linear or Cox models estimating the association of residential segregation with later life cognitive abilities or incident dementia. Estimates for which the 95% confidence intervals do not include the null are highlighted in bold.

Residential Segregation Indices ^1^	Memory ^2^ β (95% CI)	Language ^2^ β (95% CI)	Visuospatial ^2^ β (95% CI)	Incident Dementia ^3^ HR (95% CI)
Dissimilarity NH Black-NH White	**−0.054 (−0.108; –0.001)**	**−0.052 (−0.099; –0.005)**	−0.037 (−0.085; 0.011)	1.14 (0.92; 1.41)
Dissimilarity Hispanic-NH White	0.027 (−0.014; 0.067)	**−0.051 (−0.087; −0.016)**	**−0.043 (−0.079; –0.007)**	0.96 (0.82; 1.13)
Isolation NH Black	−0.049 (−0.104; 0.007)	**−0.053 (−0.101; −0.006)**	−0.043 (−0.093; 0.006)	1.16 (0.94; 1.44)
Isolation Hispanic	0.013 (−0.029; 0.055)	**−0.048 (−0.084; −0.013)**	**−0.054 (−0.090; –0.019)**	1.07 (0.91; 1.26)
Isolation NH White	0.004 (−0.056; 0.065)	**0.120 (0.070; 0.170)**	0.040 (−0.010; 0.091)	0.86 (0.62; 1.19)
Interaction NH Black-NH White	0.015 (−0.027; 0.058)	**0.061 (0.026; 0.095)**	0.012 (−0.026; 0.050)	0.94 (0.78; 1.14)
Interaction Hispanic-NH White	**0.062 (0.020; 0.105)**	**0.067 (0.032; 0.103)**	**0.050 (0.014; 0.087)**	0.94 (0.79; 1.12)

^1^ Racial/ethnic residential segregation indices are measured at the block group level from 2005–2009 ACS data. Dissimilarity measures the number of people who would have to move to create an equal distribution of a racial/ethnic group in the geographic area of interest. Isolation and interaction measure the likelihood of interacting with someone in the same racial/ethnic group or in a different racial/ethnic group, respectively. For all indices, we used the mean value within our sample to create binary indicators of segregated census blocks. Higher dissimilarity and isolation indicate a block group with more people from minoritized backgrounds, whereas higher interaction indicates more desegregated areas.; ^2^ multilevel linear models adjusted for age, sex/gender, race/ethnicity, cSES, years of education, occupation, language of test administration, birthplace, and recruitment cohort. All cognitive scores were converted to z-scores; ^3^ multilevel Cox models with age as the underlying time-scale, adjusted for sex/gender, race/ethnicity, childhood socioeconomic position (cSES), years of education, occupation, language of test administration, birthplace, and recruitment cohort; Abbreviations: CI, confidence interval; HR, hazard ratio; NH, non-Hispanic.

**Table 3:** Results of multilevel linear or Cox models with an interaction between segregation index and race/ethnicity estimating the association of residential segregation with later life cognitive abilities among respondents from different groups. Estimates for which the 95% confidence intervals do not include the null are highlighted in bold.

Residential Segregation ^1^ Indices	Memory ^2^	Language ^2^	Visuospatial ^2^	Incident Dementia ^3^
	β (95% CI)	β (95% CI)	β (95% CI)	HR (95% CI)
**NH Black respondents**				
*Dissimilarity NH Black—NH White*	**−0.073 (−0.143; −0.003)**	**−0.071 (−0.129; −0.012)**	−0.008 (−0.067; 0.051)	**1.74 (1.34; 2.27)**
*Dissimilarity Hispanic-NH White*	0.032 (−0.039; 0.103)	−0.040 (−0.101; 0.021)	−0.042 (−0.103; 0.018)	1.05 (0.78; 1.41)
*Isolation NH Black*	−0.053 (−0.121; 0.015)	**−0.074 (−0.132; −0.017)**	−0.017 (−0.075; 0.042)	**1.72 (1.32; 2.24)**
*Isolation Hispanic*	0.032 (−0.040; 0.104)	−0.010 (−0.072; 0.052)	−0.040 (−0.100; 0.020)	1.27 (0.95; 1.69)
*Isolation NH White ^4^*	0.093 (−0.069; 0.255)	**0.140 (0.019; 0.262)**	0.093 (−0.033; 0.219)	1.01 (0.32; 3.19)
*Interaction NH Black—NH White*	0.028 (−0.042; 0.098)	0.044 (−0.012; 0.100)	0.045 (−0.014; 0.104)	1.21 (0.91; 1.61)
*Interaction Hispanic—NH White*	0.087 (−0.007; 0.180)	0.066 (−0.010; 0.142)	0.004 (−0.072; 0.080)	0.82 (0.50; 1.34)
**Hispanic respondents**				
*Dissimilarity NH Black-NH White*	−0.015 (−0.107; 0.077)	−0.027 (−0.101; 0.048)	−0.066 (−0.142; 0.010)	1.05 (0.76; 1.44)
*Dissimilarity Hispanic-NH White*	0.020 (−0.038; 0.078)	−0.034 (−0.082; 0.014)	**−0.050 (−0.098; –0.002)**	1.05 (0.86; 1.28)
*Isolation NH Black*	−0.029 (−0.125; 0.067)	−0.024 (−0.101; 0.054)	**−0.086 (−0.166; –0.007)**	1.05 (0.76; 1.46)
*Isolation Hispanic*	−0.009 (−0.068; 0.050)	−0.041 (−0.089; 0.007)	**−0.079 (−0.126; –0.031)**	1.13 (0.93; 1.39)
*Isolation NH White*	0.048 (−0.065; 0.162)	**0.095 (0.006; 0.185)**	0.071 (−0.020; 0.162)	0.87 (0.54; 1.38)
*Interaction NH Black-NH White*	0.030 (−0.040; 0.101)	0.043 (−0.012; 0.099)	0.030 (−0.029; 0.089)	1.04 (0.81; 1.33)
*Interaction Hispanic—NH White*	**0.060 (0.002; 0.119)**	**0.076 (0.029; 0.123)**	**0.080 (0.031; 0.128)**	0.95 (0.78; 1.17)
**NH White respondents**				
*Dissimilarity NH Black-NH White ^4^*	–0.096 (–0.308; 0.115)	−0.016 (−0.192; 0.159)	−0.164 (−0.330; 0.002)	0.98 (0.24; 3.98)
*Dissimilarity Hispanic-NH White*	0.034 (–0.054; 0.121)	**−0.114 (−0.187; –0.041)**	−0.028 (−0.099; 0.044)	**0.53 (0.34; 0.84)**
*Isolation NH Black ^4^*	–0.185 (–0.507; 0.137)	0.068 (−0.182; 0.318)	−0.166 (−0.414; 0.083)	N/A
*Isolation Hispanic*	0.036 (–0.053; 0.124)	**−0.122 (−0.196; –0.049)**	−0.018 (−0.089; 0.053)	**0.60 (0.38; 0.94)**
*Isolation NH White*	–0.038 (–0.114; 0.038)	**0.126 (0.063; 0.190)**	0.013 (−0.050; 0.076)	**0.52 (0.35; 0.78)**
*Interaction NH Black-NH White*	–0.017 (–0.094; 0.059)	**0.099 (0.038; 0.159)**	−0.044 (−0.107; 0.019)	**0.50 (0.31; 0.83)**
*Interaction Hispanic-NH White*	0.049 (–0.031; 0.128)	0.051 (−0.013; 0.116)	0.029 (−0.036; 0.094)	**0.58 (0.42; 0.80)**

^1^ Racial/ethnic residential segregation indices are measured at the block group level from 2005–2009 ACS data. Dissimilarity measures the number of people who would have to move to create an equal distribution of a racial/ethnic groups in the geographic area of interest. Isolation and interaction measure the likelihood of interacting with someone in the same racial/ethnic group or in a different racial/ethnic group, respectively. For all indices, we used the mean value within our sample to create binary indicators of segregated census blocks. Higher dissimilarity and isolation indicate a block group with more people from minoritized backgrounds, whereas higher interaction indicates more desegregated areas; ^2^ multilevel linear models adjusted for age, sex/gender, race/ethnicity, cSES, years of education, occupation, language of test administration, birthplace, and recruitment cohort. All cognitive scores were converted to z-scores; ^3^ multilevel Cox models with age as the underlying time-scale, adjusted for sex/gender, race/ethnicity, childhood socioeconomic position (cSES), years of education, occupation, language of test administration, birthplace, and recruitment cohort; ^4^ due to stark segregation between NH Black and NH White adults in the studied area, very few (*n* = 70) of our NH Black respondents live in a census block where the majority of residents are NH White adults, as indicated by the isolation NH White index. Similarly, very few NH White respondents live in census blocks where the majority of residents are NH Black as indicated by the dissimilarity NH Black-NH White index (*n* = 43) and isolation NH Black index (*n* = 14). Subsequently, the concerned estimates have wide confidence intervals; abbreviations: CI, confidence interval; HR, hazard ratio; NH, non-Hispanic.

## Data Availability

The data that support these findings are available with approved data request (https://cumc.co1.qualtrics.com/jfe/form/SV_6x5rRy14B6vpoqNM, accessed on 1 June 2020). Study protocol, data management, and statistical analysis coding will be shared upon request from the corresponding author of this study.

## Supplementary Materials

The following are available online at https://www.mdpi.com/article/10.3390/ijerph182111233/s1, Material S1: Equations for segregation indices, Figure S1: Study population flowchart for cognitive outcomes, Figure S2: Study population flowchart for dementia analysis, Figure S3: Maps of additional segregation indices, Table S1: Descriptive statistics of those with and without missing data, Table S2: Proportion in poverty across segregation indices, Table S3: Results of models adjusted for age, race/ethnicity, and sex/gender, Table S4: Results of models adjusted also for possible mediators, Table S5: Results of models employing multiple imputation. Table S6: P-values for the interaction term between segregation index & race/ethnicity, Table S7: Results of language models adjusted for number of depressive~symptoms.
