# Supplementary material for: Relationship between Residential Segregation, Later-Life Cognition, and Incident Dementia across Race/Ethnicity
Source: Int J Environ Res Public Health. 2021 Oct 26;18(21):11233. doi: 10.3390/ijerph182111233 (PMC8583156; doi:10.3390/ijerph182111233)
Supplement: Supplementary file 1 [file ijerph-18-11233-s001.zip › ijerph-1381592-supplementary.pdf]

Supplemental materials for manuscript "Relationship between Residential Segregation, Later-Life Cognition, and Incident Dementia Across Race/Ethnicity"

Supplementary Material S1. Equations for segregation indices

Here we detail how racial/ethnic residential segregation indices were calculated using NH Black and NH White groups as an example. For Hispanic-NH White Dissimilarity, Hispanic-NH White Interaction and Hispanic Isolation, the number of Hispanic residents in a block group was used. In their original forms, these indices summate the values calculated for each smaller geographic unit (e.g., block group) in order to provide an overall segregation value for the larger geographic unit (e.g. census tract). This allows for an understanding of the segregation status of the entire geographic region of interest (e.g., Washington Heights/Inwood, New York). For example, for dissimilarity, if the sum of all the values calculated for each block group is equal to 0, then the larger geographic unit (Washington Heights/Inwood) as a whole is not considered to be segregated. By contrast, with our calculations, we determine the degree of segregation of each block group. We cannot determine how segregated Washington Heights actually is without aggregating the block group values; however, this is ideal for our study, as it does not limit our ability to focus on a smaller geographic unit, a necessity when studying such a diverse and densely populated region. Note: Interaction and Isolation are not inverse of each other in our study, because there are multiple racial/ethnic groups in each areal unit.

$$Dissimilarity = \frac{w_i}{W_T} - \frac{b_i}{B_T}$$

$w_i = number\ of\ NH\ Whites\ in\ block\ group\ i$

$W_T = total\ number\ of\ NH\ Whites\ in\ the\ New\ York\ City\ borough\ of\ interest$

$b_i = number\ of\ NH\ Blacks\ in\ block\ group\ i$

$B_T = total\ number\ of\ NH\ Blacks\ in\ the\ New\ York\ City\ borough\ of\ interest$

$$Interaction = (\frac{b_i}{B_T})(\frac{w_i}{N_i})$$

$b_i = number\ of\ NH\ Blacks\ in\ block\ group\ i$

$B_T = total\ number\ of\ NH\ Blacks\ in\ the\ New\ York\ City\ borough\ of\ interest$

$w_i = number\ of\ NH\ Whites\ in\ block\ group\ i$

$N_i = total\ population\ of\ block\ group\ i$

$$Isolation = (\frac{b_i}{B_T})(\frac{b_i}{N_i})$$

$b_i = number\ of\ NH\ Blacks\ in\ block\ group\ i$

$B_T = total\ number\ of\ NH\ Blacks\ in\ the\ New\ York\ City\ borough\ of\ interest$

$N_i = total\ population\ of\ block\ group\ i$

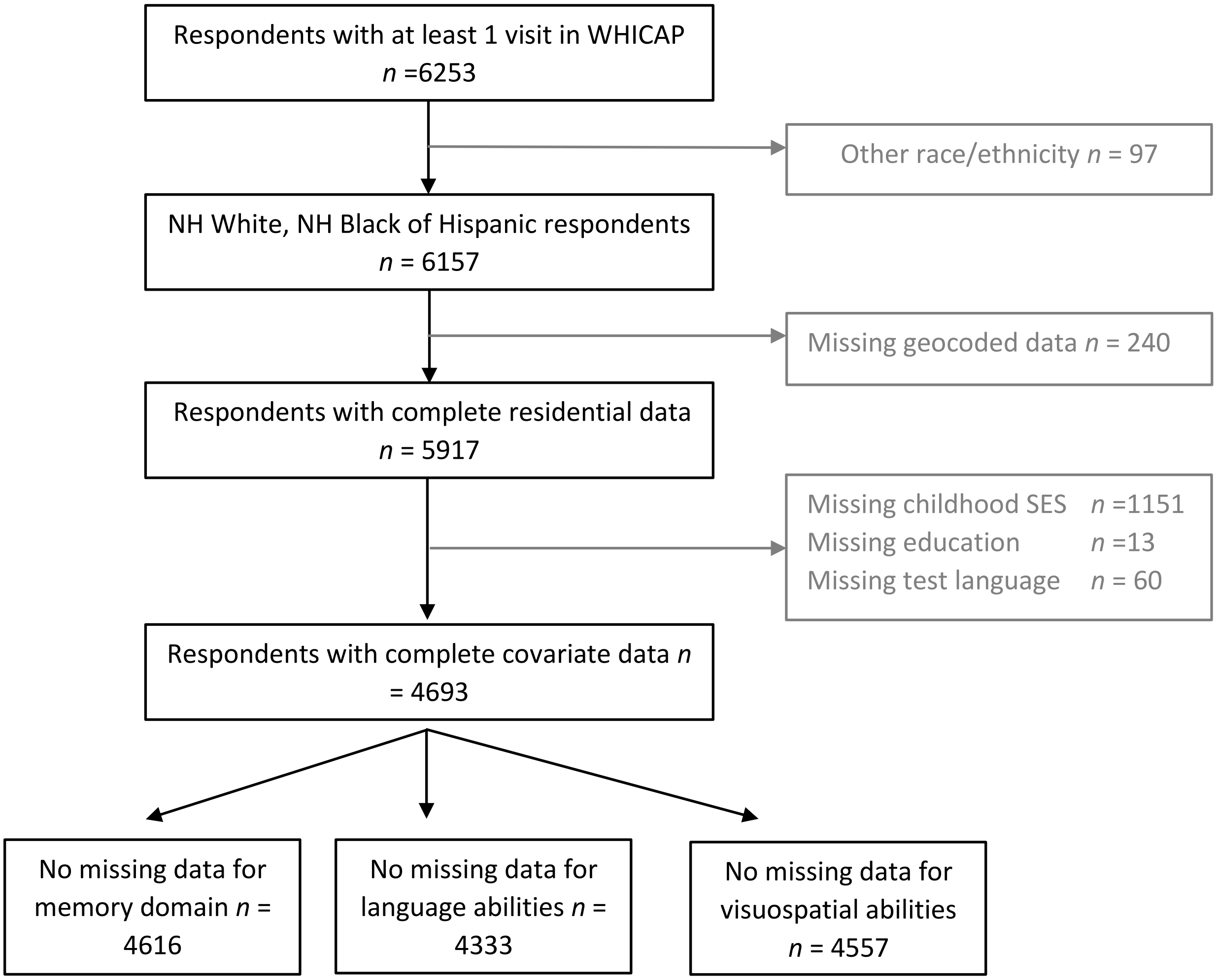

Figure S1. Study population flow chart for cognitive outcomes

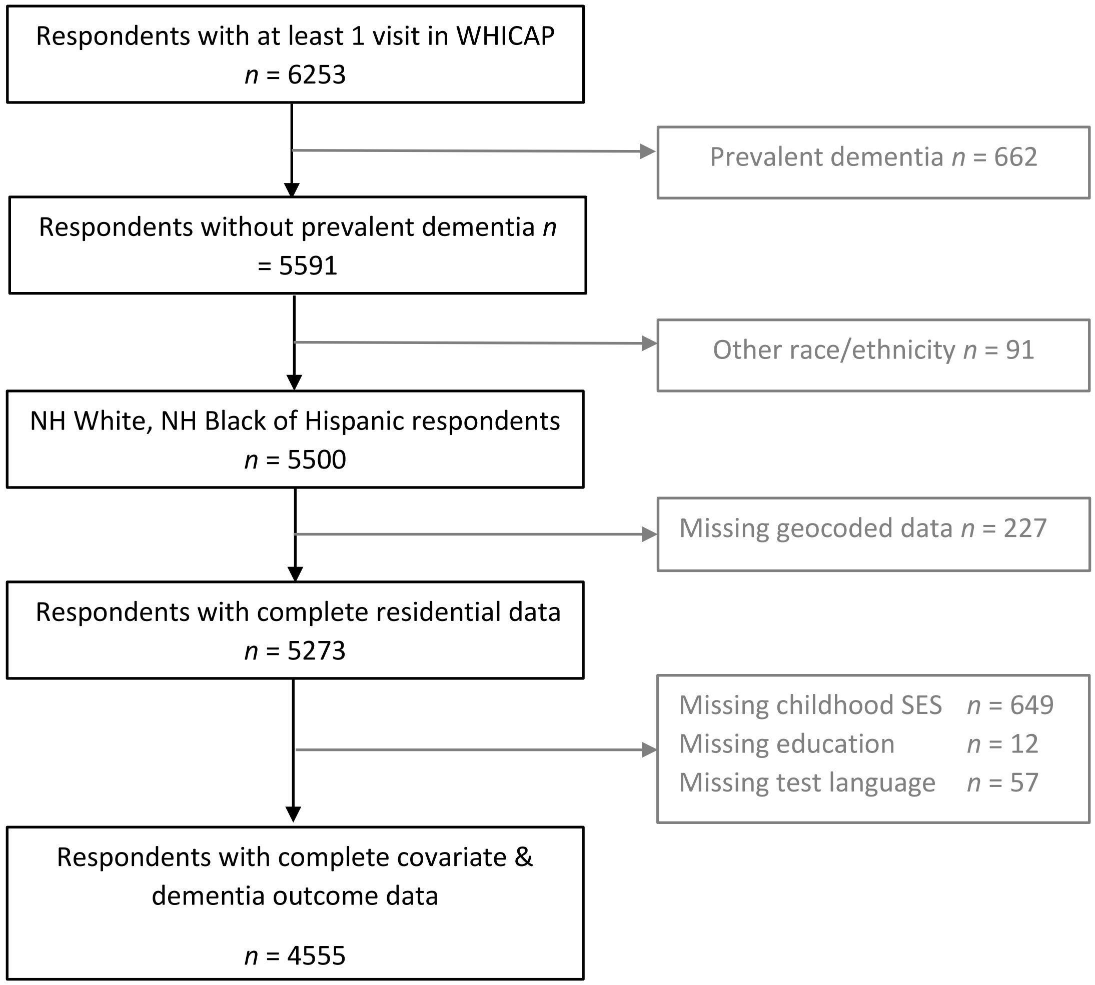

Figure S2. Study population flow chart for dementia analysis

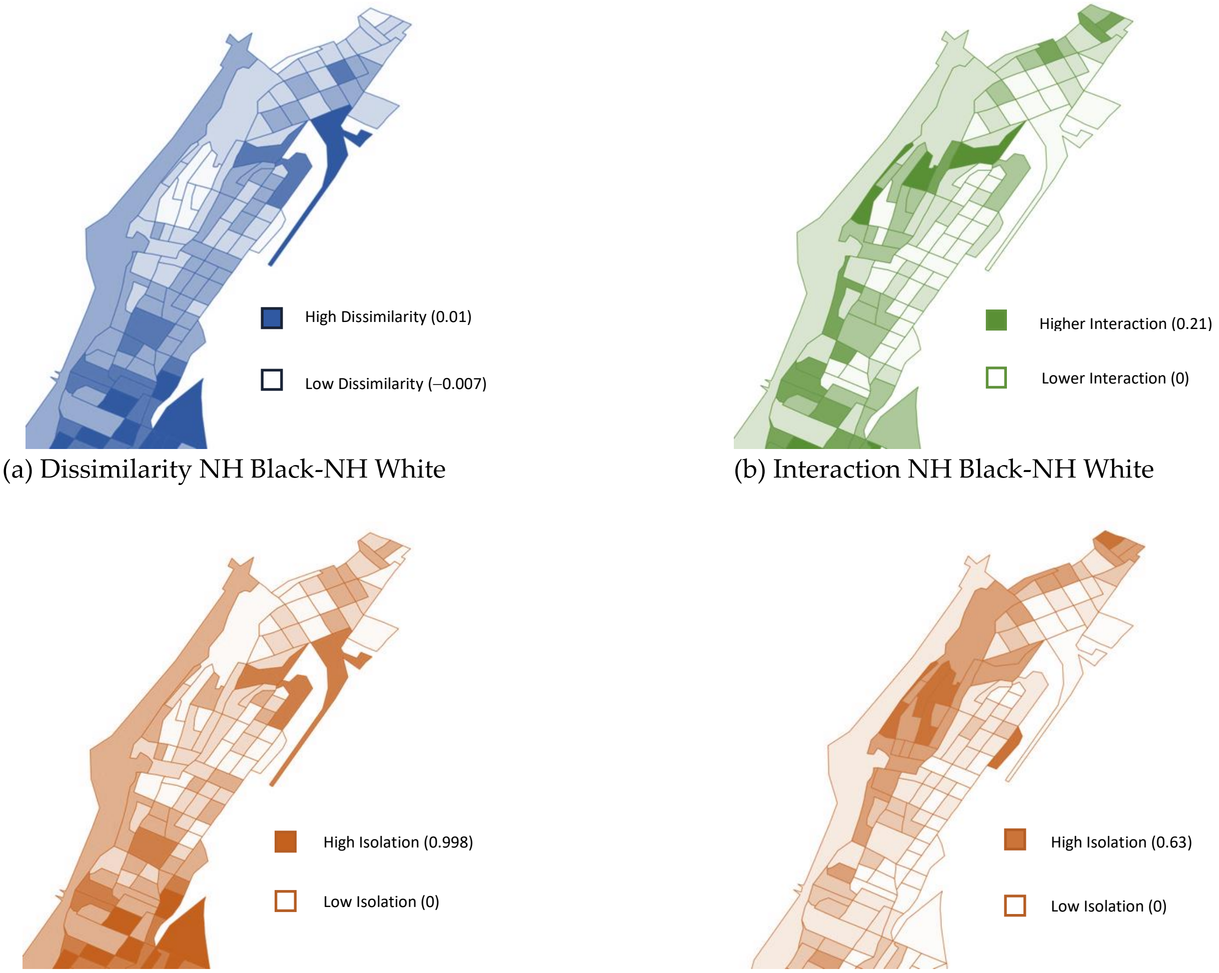

(a) Dissimilarity NH Black-NH White

(b) Interaction NH Black-NH White

(c) Isolation NH Black

(d) Isolation NH White

Figure S3. Maps of additional segregation indices

This figure demonstrates the unique information provided by the three different racial residential segregation indices (dissimilarity, interaction, isolation). The maps below show the block groups for Washington Heights and Inwood, New York. (**a**) Dissimilarity index for people who are non-Hispanic Black and non-Hispanic White. Darker blue is higher dissimilarity; lighter blue is lower dissimilarity. (**b**) Interaction index for people who are non-Hispanic Black and non-Hispanic White. Darker green is higher interaction; lighter green is lower interaction. (**c**) Isolation index for people who are non-Hispanic Black. Darker orange is higher isolation; lighter orange is lower isolation. (d) Isolation index for people who are non-Hispanic White.

Table S1. Descriptive statistics for those with and without missing covariate data.

	No missing covariate n = 4693	Missing covariate data n = 1224	t/ χ^2	*p*-value
**Segregation indices**				
Segregated area, No. (%)	2263 (48.2%)	669 (54.7%)	16.1	<0.0001
High Dissimilarity Black-White, No. (%)	1138 (24.3%)	321 (26.2%)	2.0	0.15
High Dissimilarity Hispanic/Latinx-White, No. (%)	2145 (45.7%)	630 (51.4%)	13.0	0.0003
High Isolation Black, No. (%)	954 (20.3%)	277 (22.6%)	3.1	0.08
High Isolation Hispanic/Latinx. No. (%)	2175 (46.4%)	638 (52.1%)	13.0	0.0003
High Isolation White, No. (%)	892 (19.0%)	142 (11.6%)	36.9	<0.0001
High Interaction Black-White, No. (%)	1726 (36.8%)	382 (31.2%)	13.1	0.0003
High Interaction Hispanic/Latinx-White, No. (%)	1750 (37.3%)	393 (32.1%)	11.3	0.0008
**Cognitive outcomes**				
Memory, mean (SD)	0.257 (0.74)	-0.439 (1.02)	25.4	<0.0001
Language abilities, mean (SD)	0.246 (0.68)	-0.310 (0.85)	20.1	<0.0001
Visuospatial abilities, mean (SD)	0.270 (0.64)	-0.142 (0.76)	16.2	<0.0001
Dementia, No. (%) [1]	737 (16.2%)	94 (13.1%)	4.5	0.03
**Sociodemographic**				
Age, mean (SD), y	75.8 (6.5)	79.0 (7.7)	13.3	<0.0001
Educational level, mean (SD), y	10.2 (5.0)	8.4 (5.1)	10.87	<0.0001
Women, No. (%)	3161 (67.4%)	841 (68.7%)	0.81	<0.37
Race/ethnicity				
% Hispanic/Latino	1173 (25.0%)	234 (19.2%)	18.6	<0.0001
% Black	1425 (30.4%)	406 (33.2%)		
% Non-Hispanic white	2095 (44.6%)	584 (47.7%)		
Occupation, No. (%)				
% Low	2370 (50.5%)	639 (52.2%)	320.9	<0.0001
% Middle	1016 (21.7%)	180 (14.7%)		
% High	1090 (23.2%)	174 (14.2%)		
% Not reported	317 (4.6%)	231 (18.9%)		
Birthplace, No. (%)				
% US	1746 (37.2%)	284 (23.2%)	199.7	<0.0001
% Puerto Rico	193 (4.1%)	29 (2.4%)		
% Dominican Republic	1162 (24.8%	228 (16.6%)		
% Not reported	1592 (33.9%)	683 (55.8%)		
English as primary language, No. (%)	2724 (58.3%)	648 (56.6%)	13.2	0.001
Cohort, No. (%)				
% 1992	1196 (25.5%)	521 (42.6%)	158.3	<0.0001
% 1998	1717 (36.6%)	417 (34.1%)		
% 2009	1780 (37.9%)	286 (23.4%)		
Neighborhood poverty level, mean (SD)	0.24 (0.13)	0.26 (0.13)	3.42	0.0006
**Clinical risk factors**				
CVD count, mean (SD)	1.2 (0.93)	1.2 (0.95)	1.24	0.022

[1] for dementia analyses there were 718 observations with missing data on covariates.

Table S2. Proportion in poverty across segregation indices.

Table S2. Descriptive statistics for proportion of living in poverty in a census block group across the segregation indices.

	Mean	SD	Min-Max
High Isolation NH White	0.12	(0.09)	0.00 - 0.40
High Interaction NH Black-NH White	0.20	(0.10)	0.00 - 0.55
High Interaction Hispanic-NH White	0.20	(0.11)	0.01 - 0.56
Low Dissimilarity Hispanic-NH White	0.21	(0.13)	0.08 - 0.61
Low Isolation Hispanic	0.21	(0.13)	0.00 - 0.88
Low Dissimilarity NH Black-NH White	0.24	(0.13)	0.00 - 0.79
Low Isolation NH Black	0.24	(0.13)	0.00 - 0.79
High Dissimilarity NH Black-NH White	0.27	(0.13)	0.00 - 0.88
Low Isolation NH White	0.27	(0.12)	0.00 - 0.88
Low Interaction NH Black-NH White	0.27	(0.13)	0.00 - 0.88
Low Interaction Hispanic-NH White	0.27	(0.13)	0.00 - 0.88
High Isolation NH Black	0.28	(0.12)	0.00 - 0.88
High Isolation Hispanic	0.28	(0.11)	0.08 - 0.61
High Dissimilarity Hispanic-NH White	0.29	(0.11)	0.00 - 0.88

Table S3.Results of models adjusted for age, race/ethnicity, and sex/gender.

Table S3. Results of multilevel linear or Cox models estimating the association of residential segregation with later life cognitive abilities, adjusted for age, race/ethnicity, and sex/gender.

**Residential Segregation Indices**[1]	**Memory**[2]	**Language**[2]	**Visuospatial**[2]	**Incident dementia**[3]
	β (95% CI)	β (95% CI)	β (95% CI)	HR (95% CI)
Dissimilarity NH Black-NH White	-0.059 (-0.127; 0.010)	-0.044 (-0.125; 0.037)	-0.018 (-0.085; 0.049)	1.048 (0.819; 1.341)
Dissimilarity Hispanic-NH White	-0.029 (-0.080; 0.022)	-0.196 (-0.256; -0.136)	-0.137 (-0.185; -0.088)	1.106 (0.926; 1.322)
Isolation NH Black	-0.067 (-0.137; 0.003)	-0.027 (-0.111; 0.056)	-0.020 (-0.089; 0.050)	1.067 (0.828; 1.376)
Isolation Hispanic	-0.037 (-0.088; 0.014)	-0.190 (-0.250; -0.130)	-0.144 (-0.193; -0.096)	1.226 (1.031; 1.459)
Isolation NH White	0.123 (0.053; 0.193)	0.363 (0.292; 0.433)	0.206 (0.143; 0.269)	0.661 (0.474; 0.921)
Interaction NH Black-NH White	0.069 (0.017; 0.122)	0.201 (0.141; 0.260)	0.105 (0.053; 0.157)	0.832 (0.676; 1.024)
Interaction Hispanic-NH White	0.089 (0.039; 0.139)	0.120 (0.058; 0.182)	0.100 (0.050; 0.149)	0.856 (0.709; 1.033)

[1] Racial/ethnic residential segregation indices are measured at the block group level from 2005-2009 ACS data. Dissimilarity measures the number of people who would have to move to create an equal distribution of a racial/ethnic groups in the geographic area of interest. Isolation and interaction measure the likelihood of interacting with someone in the same racial/ethnic group or in a different racial/ethnic group, respectively. For all indices, we used the mean value within our sample to create binary indicators of segregated census blocks. Higher dissimilarity and isolation indicate a block group with more people from minoritized backgrounds, whereas higher interaction indicates more desegregated areas. [2]Multilevel linear models adjusted for age, sex/gender, race/ethnicity. All cognitive scores were converted to z-scores. [3] Multilevel Cox models with age as the underlying time-scale, adjusted for sex/gender, race/ethnicity. Abbreviations: CI, confidence interval; HR, hazard ratio; NH, non-Hispanic.

Table S4. Results models adjusted for possible mediators (area poverty & CVD)

Table S4. Results of multilevel linear or Cox models estimating the association of residential segregation with later life cognitive abilities, adjusted for age, sex/gender, race/ethnicity, childhood SES, years of education, occupation, language of test administration, birthplace, recruitment cohort, CVD count and proportion living in poverty in a census block.

Residential Segregation Indices[1]	Memory[2]	Language[2]	Visuospatial[2]	Incident dementia[3]
	β (95%CI)	β (95%CI)	β (95%CI)	HR (95%CI)
Dissimilarity NH Black-NH White	-0.053 (-0.107; 0.001)	-0.046 (-0.093; -0.001)	-0.034 (-0.082; 0.014)	1.129 (0.912; 1.397)
Dissimilarity Hispanic-NH White	0.030 (-0.012; 0.072)	-0.041 (-0.077; -0.006)	-0.039 (-0.076; -0.002)	0.944 (0.802; 1.111)
Isolation NH Black	-0.049 (-0.105; 0.007)	-0.047 (-0.094; 0.000)	-0.040 (-0.090; 0.009)	1.143 (0.924; 1.413)
Isolation Hispanic	0.016 (-0.027; 0.058)	-0.040 (-0.075; -0.004)	-0.051 (-0.086; -0.015)	1.061 (0.902; 1.248)
Isolation NH White	0.001 (-0.062; 0.065)	0.107 (0.054; 0.160)	0.032 (-0.021; 0.086)	0.900 (0.646; 1.254)
Interaction NH Black-NH White	0.016 (-0.028; 0.059)	0.054 (0.020; 0.088)	0.009 (-0.029; 0.048)	0.960 (0.795; 1.160)
Interaction Hispanic-NH White	0.064 (0.022; 0.107)	0.063 (0.029; 0.098)	0.049 (0.013; 0.086)	0.947 (0.794; 1.129)

[1] Racial/ethnic residential segregation indices are measured at the block group level from 2005-2009 ACS data. Dissimilarity measures the number of people who would have to move to create an equal distribution of a racial/ethnic groups in the geographic area of interest. Isolation and interaction measure the likelihood of interacting with someone in the same racial/ethnic group or in a different racial/ethnic group, respectively. For all indices, we used the mean value within our sample to create binary indicators of segregated census blocks. Higher dissimilarity and isolation indicate a block group with more people from minoritized backgrounds, whereas higher interaction indicates more desegregated areas. [2]Multilevel linear models adjusted for age, sex/gender, race/ethnicity, childhood SES, years of education, occupation, language of test administration, birthplace, recruitment cohort, CVD count and proportion living in poverty in a census block. All cognitive scores were converted to z-scores. [3] Multilevel Cox models with age as the underlying time-scale, adjusted for sex/gender, race/ethnicity. Abbreviations: CI, confidence interval; HR, hazard ratio; NH, non-Hispanic.

Table S5. Results of models employing multiple imputation

Table S5. Results of multilevel linear or Cox models estimating the association of residential segregation with later life cognitive abilities or incident dementia when multiple imputation (10 datasets) using Markov chain Monte Carlo method is used. Estimates for which the 95% confidence intervals do not include the null are highlighted in bold.

Residential Segregation Indices[1]	Memory[2]	Language[2]	Visuospatial[2]	Incident dementia[3]
	β (95%CI)	β (95%CI)	β (95%CI)	HR (95%CI)
Dissimilarity NH Black-NH White	-0.054 (-0.111; 0.003)	**-0.058 (-0.104; -0.012)**	**-0.049 (-0.097; -0.001)**	**1.25 (1.01; 1.54)**
Dissimilarity Hispanic-NH White	0.030 (-0.013; 0.074)	-0.029 (-0.063; 0.005)	-0.027 (-0.063; 0.010)	0.94 (0.80; 1.10)
Isolation NH Black	**-0.058 (-0.116; -0.000)**	**-0.056 (-0.103; -0.009)**	**-0.053 (-0.102; -0.004)**	**1.29 (1.05; 1.57)**
Isolation Hispanic	0.020 (-0.024; 0.065)	-0.021 (-0.055; 0.013)	-0.035 (-0.072; 0.001)	0.99 (0.84; 1.16)
Isolation NH White	-0.002 (-0.067; 0.063)	**0.123 (0.073; 0.172)**	0.043 (-0.008; 0.093)	0.93 (0.69; 1.27)
Interaction NH Black-NH White	0.012 (-0.032; 0.057)	**0.064 (0.032; 0.096)**	0.016 (-0.021; 0.053)	0.96 (0.80; 1.15)
Interaction Hispanic-NH White	**0.059 (0.016; 0.103)**	**0.086 (0.052; 0.119)**	**0.074 (0.039; 0.109)**	1.00 (0.84; 1.19)

[1] Racial/ethnic residential segregation indices are measured at the block group level from 2005-2009 ACS data. Dissimilarity measures the number of people who would have to move to create an equal distribution of a racial/ethnic groups in the geographic area of interest. Isolation and interaction measure the likelihood of interacting with someone in the same racial/ethnic group or in a different racial/ethnic group, respectively. For all indices, we used the mean value within our sample to create binary indicators of segregated census blocks. Higher dissimilarity and isolation indicate a block group with more people from minoritized backgrounds, whereas higher interaction indicates more desegregated areas. [2]Multilevel linear models adjusted for age, sex/gender, race/ethnicity, childhood SES, years of education, occupation, language of test administration, birthplace, recruitment cohort, CVD count and proportion living in poverty in a census block. All cognitive scores were converted to z-scores. [3] Multilevel Cox models with age as the underlying time-scale, adjusted for sex/gender, race/ethnicity. Abbreviations: CI, confidence interval; HR, hazard ratio; NH, non-Hispanic.

Table S6. P-values for the interaction term between segregation index & race/ethnicity

Table S6. P-values for the interaction between segregation index and race/ethnicity in multilevel linear or Cox models estimating the association of residential segregation with later life cognitive abilities among respondents from racial/ethnic different groups. The bolded values highlight p-values smaller than 0.2 [1]

Residential Segregation Indices	Memory[2]	Language[2]	Visuospatial[2]	Incident dementia[3]
	p-value	*p*-value	*p*-value	*p*-value
Dissimilarity NH Black-NH White	0.56	0.57	0.13	0.86
Dissimilarity Hispanic-NH White	0.96	0.16	0.87	0.01
Isolation NH Black	0.65	0.34	0.21	0.54
Isolation Hispanic	0.59	0.06	0.30	0.04
Isolation NH White	0.23	0.80	0.37	0.38
Interaction NH Black-NH White	0.60	0.32	0.09	0.73
Interaction Hispanic-NH White	0.83	0.82	0.17	0.48

[1] Differences across groups were interpreted based on magnitude of estimates and their confidence intervals, in addition to considering p-value for the interaction term. [2] Multilevel linear models adjusted for age, sex/gender, race/ethnicity, cSES, years of education, occupation, language of test administration, birthplace, and recruitment cohort. All cognitive scores were converted to z-scores. [3] Multilevel Cox models with age as the underlying time-scale, adjusted for sex/gender, race/ethnicity, childhood socioeconomic position (cSES), years of education, occupation, language of test administration, birthplace, and recruitment cohort. Abbreviations: CI, confidence interval; HR, hazard ratio; NH, non-Hispanic.

Table S7. Results of language models adjusted for number of depressive symptoms.

Table S7. Results of multilevel linear models estimating the association of residential segregation with later life language abilities. Comparing results of model 3 that include two mediators (CVD count and proportion living in poverty in a census block) and employs multiple imputation and model 4 that added number of depressive symptoms and employs multiple imputation.

Residential Segregation Indices[1]	Language[2] (M3; Multiple imputation)	Language[3] (M4 =M3 + CESD; Multiple imputation)
	β (95%CI)	β (95%CI)
Dissimilarity Black-White	-0.043 (-0.087; 0.001)	-0.042 (-0.085; 0.002)
Dissimilarity Hispanic/Latinx-White	-0.019 (-0.052; 0.013)	-0.019 (-0.051; 0.014)
Isolation Black	-0.041 (-0.086; 0.005)	-0.039 (-0.08; 0.007)
Isolation Hispanic/Latinx	-0.012 (-0.045; -0.02)	-0.011 (-0.043; -0.022)
Isolation White	0.102 (0.051; 0.154)	0.100 (0.048; 0.151)
Interaction Black-White	0.058 (0.027; 0.090)	0.0556 (0.024; 0.087)
Interaction Hispanic/Latinx-White	0.074 (0.040; 0.107)	0.074 (0.041; 0.108)

[1] Racial/ethnic residential segregation indices are measured at the block group level from 2005-2009 ACS data. Dissimilarity measures the number of people who would have to move to create an equal distribution of a racial/ethnic groups in the geographic area of interest. Isolation and interaction measure the likelihood of interacting with someone in the same racial/ethnic group or in a different racial/ethnic group, respectively. For all indices, we used the mean value within our sample to create binary indicators of segregated census blocks. Higher dissimilarity and isolation indicate a block group with more people from minoritized backgrounds, whereas higher interaction indicates more desegregated areas. [2]Multilevel linear models adjusted for age, sex/gender, race/ethnicity, childhood SES, years of education, occupation, language of test administration, birthplace, recruitment cohort, CVD count and proportion living in poverty in a census block. Language scores were converted to z-scores. [3] The model added depressive symptoms (measured using the short version of the Center for Epidemiologic Studies-Depression Scale) as a covariate. Language scores were converted to z-scores. Abbreviations: CI, confidence interval; HR, hazard ratio; NH, non-Hispanic.
